# The Blood and Muscle Expression Pattern of the Equine *TCAP* Gene during the Race Track Training of Arabian Horses

**DOI:** 10.3390/ani9080574

**Published:** 2019-08-18

**Authors:** Monika Stefaniuk-Szmukier, Tomasz Szmatoła, Joanna Łątka, Bogusława Długosz, Katarzyna Ropka-Molik

**Affiliations:** 1 Department of Animals Reproduction, Anatomy and Genomics, the University of Agriculture in Krakow, Al. Mickiewicza 24/28, 30-159 Kraków, Poland; 2 Department of Animal Molecular Biology, National Research Institute of Animal Production, Krakowska 1, 32-083 Balice, Poland; 3 Centre of Veterinary Medicine, University of Agriculture in Kraków, Al. Mickiewicza 24/28, 30-059 Kraków, Poland

**Keywords:** TCAP, muscle, Arabian horses, gene expression, training exercise

## Abstract

**Simple Summary:**

The highly selective breeding of horses results in a variety of fiber type compositions among the breeds. Moreover, fiber type composition is known to differ within and between muscles. Telethonin (*TCAP*) plays a significant role in myofibril assembly, muscle development, and functional regulation. Analysis performed by qPCR of the gluteus medius muscle and the whole blood of Arabian horses during the training schedule showed that the expression pattern of the *TCAP* gene differs between skeletal muscles and whole blood. The results of these studies are a base for further research focused on the identification of processes related to adaptation of exertion in horses.

**Abstract:**

Horse musculature has been shaped through evolution by environmental and human factors, which has resulted in several extraordinary adaptations to physical effort. Skeletal muscle plasticity results from the response to mechanical stimulation causing hypertrophy, where sarcomeres increase the muscle’s cross-sectional area under the influence of contractile forces. The aim of the present study was the evaluation of transcript abundance of the telethonin (*TCAP*) gene, which is a part of the sarcomere macromolecular mechanosensory complex in the gluteus medius muscle, and the whole blood of Arabian horses during flat race training. The analysis, performed by quantitative PCR, showed an increase of *TCAP* transcripts in skeletal muscle. However, in whole blood, the transcript abundance decreased after the first stage of training and further increased after the second phase. The obtained results indicate a lack of similarity of *TCAP* gene expression in both tissues.

## 1. Introduction

The advances of next-generation sequencing technology have revolutionized the search for molecular markers involved in several different mechanisms in the field of animal breeding [1]. In horses, previous research has led to the identification of variants associated with some utility traits; for example, a marker predicting optimal race distance in thoroughbreds has been found within the myostatin gene (*MSTN*) [2] and variation within the doublesex and mab-3-related transcription factor 3 gene (*DMRT3*) is responsible for the ability to use alternative gaits in gaited breeds [3]. By using one of the approaches of next-generation sequencing, whole transcriptome sequencing (RNA-Seq), we demonstrated that exercise regime in horses activated several pathways associated with cell cycle, communication, proliferation differentiation, and apoptosis, as well as pathways involved in peroxisome proliferator-activated receptor (PPAR) signaling, calcium signaling, and metabolic processes [4,5]. These findings led to further investigation and evaluation of RNA-Seq-based single nucleotide polymorphisms, located within the most significant deregulated differential expressed genes (DEGs), as potential markers for racing abilities in Arabian horses. The genes included *SH3RF2* (SH3 domain containing ring finger 2) [6], *ACTN3* (actinin alpha-3) [7], and *SLC16A6* (monocarboxylate transporter 7) [8,9]. The search for blood-based markers related to performance traits holds interest for many researchers. Blood-based markers, which have the potential for early detection of homeostasis imbalance, especially in connection to the musculoskeletal system, are of great importance in the equine industry. 

The highly developed horse musculature is a result of an evolutionary adaptation to speed and/or endurance. The ability to quickly run away from a predator, as well as traverse long distances, has enhanced horse adaptation to extraordinary physical effort. As a unique athlete, the horse possesses several features that enhance its athleticism, such as a high muscle mass compared to the total body weight [10]. The muscle response to conditioning is to increase muscle mass rather than the number of fibers. Selective breeding results in a variety of fiber type compositions among the breeds [11] and, furthermore, fiber type composition differs within and between the muscles [12]. Further, skeletal muscles can adapt to mechanical stimuli. Myosin, which is the main contractile protein, switches between isoform types in order to accurately generate force. This process is well recognized in horses [13,14]. Under mechanical stimulation caused by elevated forces involved during training, the force-producing units of muscle, the sarcomeres, increase the muscle cross-sectional area, leading to hypertrophy [15]. Skeletal muscle is a highly plastic tissue that is able to effectively respond to numerous metabolic and physiological stressors that vary in intensity and duration. Previous findings suggest that telethonin plays a significant role in myofibril assembly, muscle development, and functional regulation [16]. Being a part of a macromolecular mechanosensory complex, it has been shown to play a role in cardiomyopathies via their interaction with genes such as *MLP* (muscle LIM protein), *MYOZ2* (myozenin 2), *ANKRD2* (ankyrin repeat domain 2), and *sAnk1* (small muscle-specific ankyrin 1) [17,18,19]. Furthermore, based on RNA-Seq data, it has been established that *TCAP* gene transcripts are significantly deregulated in muscles and whole blood in horses under different training regimes [4,5].

Understanding the possible role of *TCAP* in working skeletal muscle is unclear. Furthermore, there are no reports analyzing the expression pattern of the telethonin (*TCAP*) gene in conditioned muscles. If the expression pattern of the *TCAP* gene correlates between tissues then the levels of *TCAP* in the blood would be informative. Thus, the aim of the present study was to investigate the changes in the transcription pattern of the *TCAP* gene in skeletal muscles and whole blood of horses that undergo physical exertion during flat race training that allows for competing in flat races.

## 2. Materials and Methods 

Animals involved in the study were pure breed Arabians of Polish origin, introduced to stud book selection under the World Arabian Horse Organization (WAHO), who had known ancestors for at least the last 150 years. A total of 23 horses were used in this study. Seven horses were sampled once when they were 2.5 years old. These horses were used as a control group and were not subjected to training or further sampling. Sixteen horses were sampled twice during the training schedule, as follows: At point A (16 horses) and again at point B (16 horses). At each time point, whole blood and tissue samples were collected from each horse. Therefore, a total of 78 biological samples were obtained, which included 39 samples of whole blood (7 + 16 + 16) and 39 samples from the gluteus medius muscle (7 + 16 + 16).

All horses were grouped according to their training schedule activity. The *TCAP* gene expression was estimated on untrained horses that were 2.5 years old (K-controls) and compared to race-trained horses (3 years old) before intensive exercise (A) and after intense effort (B).

The sampling was performed before and during a one-year period where horses were trained to achieve heightened fitness in order to compete in flat races. The 2.5-year-old animals (autumn) were (K) directed to the race track facilities for training purposes. The training schedule (seven months) included the training to be ridden and the conditioning phase with slow canters interspersed by walking or trotting. The low-speed canter distance of this phase increased to 5000 m and was followed by a decrease in high canter distance to 2500–3000 m (A). The third stage of training included an extended gallop distance, week by week, from 200 to 1200 m. Horses in training galloped distances no longer than 2000 m prior to the start of the racing season (May) (B). The sampling of whole blood and gluteus medius muscles were performed in the morning of the same day, bypassing the morning feeding due to sedation. All horses were maintained under the same environmental and feeding conditions. Diets were based on hay and oats, with a commercial feed mixture supplementation in accordance with the stage of the training cycle.

The whole blood samples were obtained by way of jugular venipuncture and collected into RNAlater tubes (Applied Biosystems, Foster City, CA, USA; ThermoFisher Scientific, Waltham, MA, USA), whereas muscle samples were collected by way of a biopsy method in accordance with procedures previously described by our team [20]. These were then stored at −80 °C. Both panels of samples were directed for further analysis.

The protocol was approved by the Animal Care and Use Committee of the Institute of Pharmacology, Polish Academy of Sciences in Cracow (no. 1173/2015).

Total RNA in the blood was extracted using a MagMAX™-96 Total RNA Isolation Kit (Ambion, John Gardiner Austin, TX, USA; Life Technologies, Carlsbad, CA, USA) according to the manufacturer’s instructions. Total RNA in the muscles was extracted by using TRIzol™ Reagent (Invitrogen, Carlsbad, CA, USA; Thermo Scientific, Waltham, MA, USA) corresponding to the protocol described by Chomczyński [21]. RNA concentration and purity were assessed using a NanoDrop 2000 Spectrophotometer (Thermo Fisher Scientific, Wilmington, DE, USA). The RNA integrity was assessed using an RNA Screen Tape and RNA Screen Tape Ladder on Tape Station 2200 (Agilent, Santa Clara, CA, USA). Samples with RIN ≥ 8.5 were reverse transcribed with the use of the High-Capacity RNA-to-cDNA Kit (Applied Biosystems, Foster City, CA, USA; Thermo Scientific, Waltham, MA, USA).

The quantitative real-time PCR (qRT-PCR) analysis was conducted using QuantStudio 7flex (Applied Biosystems) with SYBR green as the fluorescent dye, in accordance with the manufacturer’s instructions (EvaGreen^®^ qPCR Mix Plus (ROX) (Novazym)). The primers for qRT-PCR were designed using the online Prime3 program (http://primer3.ut.ee/). All the primers used in this study are listed in Table 1. All qRT-PCR reactions were conducted in triplicate with corresponding negative controls. Using the GAPDH gene for normalization, the expression pattern and qPCR efficiency were calculated in accordance with the ∆∆Ct method by Pfaffl [22].

The normality of the distribution in selected groups was checked with a Shapiro–Wilk test. Following this, the differences in the expression levels between groups were analyzed (using R software) with a Mann–Whitney test [23]. The *p*-value at <0.05 was considered as significant.

## 3. Results

**Figure 1:**
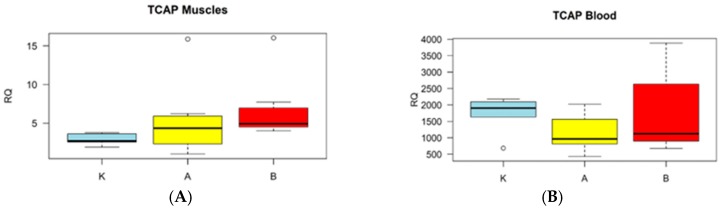
Comparison of the expression pattern of the *TCAP* gene between investigated tissues. The *TCAP* gene expression profiles (RQ) in two analyzed tissues: (**A**) Gluteus medius muscle and (**B**) whole blood. K; A; B refers to the groups at sampling points. K—Control group; A—The low-speed canter phase; B—High speed canter phase. Data are presented as means (± standard error).

Performed analysis showed the differences in the expression pattern of the *TCAP* gene between investigated tissues of Arabian horses during training periods, characterized by the intensity of exercises. The transcript abundance in skeletal muscle increased with increased effort; however, significant values have been found between the control group (K) and horses after the intense training phase (B) (*p* < 0.001). In the whole blood, the higher expression was found in a stage where heavy canters were introduced. Interestingly, the lower expression was detected after moderate training when individuals were adapted to further intensive fitness, but with no statistical significance. Obtained results also showed that the transcript abundance patterns in both tissues in the same phases are not similar (see Figure 1). There were significant differences in RQ (relative quantification) of the *TCAP* gene between the investigated tissues at the same points (K vs. K: *p* < 0.007; A vs. A: *p* < 0.0001; B vs. B: *p* < 0.00001). 

## 4. Discussion

The RNA-Seq method is widely used in searching for genetic determinants of performance in horses [2,4,5]. The global gene expression comparison of tissues sampled in different points during the training of Arabian horses allows for the marking of potential genes that, further validated, may act as potential markers of a wide range of traits [6,7]. In the present manuscript, we performed exact transcript abundance analysis in a gene previously significantly deregulated in the results of RNA-Seq analysis, in a panel of muscle and whole blood samples, for the investigation of its potential as a marker. Sarcomeres are contractile units of skeletal muscle, localized between two Z-discs. The interdigitation of thick filaments of myosin and thin filaments of actin are responsible for force generation [24]. Besides the main components of a sarcomere, the Z line is also the anchorage site for titin, the giant protein which spans nearly half of the sarcomere from the M-band to the Z-band. The Z line allows large elongation and passive force production (mechanical roles in cardiac and skeletal muscles) to take part in structural and developmental sarcomere organization and td signaling [25]. Within the Z-disc, the assembly of two titin molecules with two N-terminal immunoglobulin-like domains (Z1Z2) is mediated by a protein called telethonin [16]. This complex was recently postulated to be critical for the Z-disc structure, being required for sarcomere integrity and muscle growth [26]. Moreover, it has been shown that telethonin interacts with MLP (muscle LIM protein), FATZ (potassium channel regulation protein Mink), MSTN (myostatin), and apoptosis-inducing factor SIVA1 [27]. The implementation of *TCAP* in apoptosis has been shown in heart muscles, via the promotion of Mdm2 mediated degradation of p53 in the nuclear compartment, under biomechanical stress [28]. Consequently, in order to evaluate telethonin mRNA expression in muscles and whole blood of exercising horses, we analyzed gluteus medius muscle and blood samples collected from horses before, during, and after conditioning for racetrack performance. In the study of Dahlqvist et al., [29] the authors tested the utility of *TCAP*, among others, as a marker of disruption of sarcomere due to the muscle damage that occurred after unaccustomed eccentric exercise. However, authors could not detect the *TCAP* protein level in the serum at any time. 

Whereas *TCAP* expression might be restricted to heart and skeletal muscle tissues, we hypothesized that the whole blood reflects the changes in overall body homeostasis; thus, the expression pattern in both investigated tissues might be comparable. However, our results showed differences in expression patterns in both tissues in the same individuals, depending on the stage of training, and suggests that in whole blood *TCAP* transcripts are detectable but are not consistent with muscle transcript levels. This might suggest another source of *TCAP* transcript in blood other than skeletal muscles, and thereafter, the cardiac muscle. The discrepancies might be a result of the different adaptation of both tissues to training and thus the different signaling regulation. There is a lack of information regarding telethonin function on striated muscles. However, research performed on a zebrafish model has shown that, during growth, the early movement-generated force plays an important role in *TCAP* expression induction during muscle and T-Tubule development. This may consequently play an important role in T-tubule formation [30]. It seems that TCAP implication in mechanosensing is via interaction with MinK by the phosphorylation of telethonin and regulation of intracellular ion flow [31]. Furthermore, there is growing evidence that *TCAP* influences myostatin expression, the main regulator of myoblast proliferation and differentiation. The overexpression on *TCAP* reduced the expression of *MSTN* [32]. 

In turn, cardiac tissue-durable endurance exercises reduced resting cardiac output with a slower heart rate and increased the strength of contraction. In addition, physiological hypertrophy does not show the abnormal organization of sarcomeres and fibers [33]. Furthermore, the cardiac Z-disc complex of titin-TCAP and MLP, plays a pivotal role in cardiac muscle stretching, with functional and mechanical coupling to the T-tubule system [34]. It has been proposed that transverse tubules are critical to excitation-contraction acting in cardiomyocytes by depolarization-mediated Ca^2+^ channels [35]. It has been shown that dysfunctional *TCAP* within the Z-disc increases the calcium sensitivity via the Calsarcin-1 and calcineurin complex, which is a mediator of hypertrophy and leads to hypertrophic cardiomyopathy [36]. Thus, we assume that the downregulated expression pattern of the *TCAP* gene in the whole blood may reflect the physiological response of cardiac tissue, preventing hypertrophy due to the increased effort.

## 5. Conclusions

This work is valuable for general purposes, indicating that the hypothesis of blood reflecting changes in particular tissue might be misleading, especially when searching for tissue-specific markers. Furthermore, our findings show differences in the expression of the *TCAP* gene in horses depending on the phase of training and tissue. The main limitation of this study is that it is impossible to perform cardiac biopsies during the training schedule in horses. The presented results allow for comparison between the transcript levels of the *TCAP* gene in two different tissues (whole blood and muscle), sampled from the same horse in different training stages, and exclude *TCAP* as a potential skeletal muscle marker reflected in whole blood. However, both analyzed tissues are essential for homeostasis balance during physiological effort. Thus, the obtained results are a base for further research focused on the identification of processes related to the adaptation to effort in horses.

## Figures and Tables

**Table 1:** Primer sequences for qRT-PCR.

Ensemble Accession Number	Gene Symbol	Primer Sequence	Amplicon Length
*ENSECAG00000011860*	*TCAP*	TCAPF:GGCTGAATGGAAGGATCTGA TCAPR:TGGTAGGGCAGCTGGTACTC	192
*ENSECAG00000022051*	*GAPDH*	GAPDHF:GCCGTAACTTCTGTGCTGTG GAPDHR:AATGAAGGGGTCATTGATGG	156

The sequence of primers used for qPCR reactions. The starters were designed based on sequences deposited in the Ensemble database.
